# Melatonin Therapy in Patients with Alzheimer’s Disease

**DOI:** 10.3390/antiox3020245

**Published:** 2014-04-10

**Authors:** Daniel P. Cardinali, Daniel E. Vigo, Natividad Olivar, María F. Vidal, Luis I. Brusco

**Affiliations:** 1 Departamento de Docencia e Investigación, Facultad de Ciencias Médicas, Pontificia Universidad Católica Argentina, Buenos Aires 1007, Argentina; E-Mail: dvigo@conicet.gov.ar; 2 Centro de Neuropsiquiatría y Neurología de la Conducta, Hospital de Clínicas “José de San Martín”, Facultad de Medicina, Universidad de Buenos Aires, Buenos Aires 1121, Argentina; E-Mails: natividad.olivar@gmail.com (N.O.); flovidal@hotmail.com (M.F.V.); brusco@neuropsiquiatria.org.ar (L.I.B.)

**Keywords:** melatonin, Alzheimer’s disease, neurodegeneration, free radicals, oxidative stress, aging, mild cognitive impairment, melatonin analogs

## Abstract

Alzheimer’s disease (AD) is a major health problem and a growing recognition exists that efforts to prevent it must be undertaken by both governmental and non-governmental organizations. In this context, the pineal product, melatonin, has a promising significance because of its chronobiotic/cytoprotective properties potentially useful for a number of aspects of AD. One of the features of advancing age is the gradual decrease in circulating melatonin levels. A limited number of therapeutic trials have indicated that melatonin has a therapeutic value as a neuroprotective drug in the treatment of AD and minimal cognitive impairment (which may evolve to AD). Both *in vitro* and *in vivo*, melatonin prevented the neurodegeneration seen in experimental models of AD. For these effects to occur, doses of melatonin about two orders of magnitude higher than those required to affect sleep and circadian rhythmicity are needed. More recently, attention has been focused on the development of potent melatonin analogs with prolonged effects, which were employed in clinical trials in sleep-disturbed or depressed patients in doses considerably higher than those employed for melatonin. In view that the relative potencies of the analogs are higher than that of the natural compound, clinical trials employing melatonin in the range of 50–100 mg/day are urgently needed to assess its therapeutic validity in neurodegenerative disorders such as AD.

## 1. Introduction

Neurodegenerative disorders are a group of chronic and progressive diseases characterized by selective and symmetric losses of neurons in cognitive, motor, or sensory systems. Alzheimer’s disease (AD) is the most clinically relevant representative of this type of disorders. Although the origin of the specific neurodegeneration in AD remains undefined, three major and frequently interrelated processes, namely free radical-mediated damage, mitochondrial dysfunction, and excitotoxicity, underlie the pathophysiological mechanisms leading to neuronal death [1].

AD has a very large impact on health. In a recent publication [2] the Alzheimer’s Disease International (ADI) organization states that 44 million people live with dementia today and that this figure will rise to 135 million people by 2050. The current economic cost of dementia is $604 billion annually. It must be noted that the new estimates are an increase of 17% on the figures published in 2009, population aging being the main driver of the projected increase. The ADI report also predicts a shift in the distribution of the global burden of dementia by 2050, with 71% of all affected people living in low- or middle-income countries. It is estimated that 10% of dementia cases may be avoided by campaigns that target obesity, hypertension, diabetes, underactivity, and smoking, as well as by education and cognitive enhancement. Thus, a growing recognition exists that efforts to prevent AD at an early stage of development must be urgently undertaken.

The regular intake of antioxidants by the elderly has been one of the strategies recommended for prevention of age-associated, free radical-mediated, neurodegenerative diseases, but the efficacy of this treatment is debated [3]. In this context, the use of melatonin as a cytoprotective agent becomes of great interest. Melatonin is a well-conserved methoxyindole found in most phyla and having remarkable cytoprotective actions in addition to chronobiotic properties. The source of circulating melatonin is the pineal gland, and a substantial amount of data support that plasma melatonin decrease is one of the features of advancing age [4]. We will briefly review some relevant aspects of melatonin’s biology as far as the neurodegenerative process is concerned. Then, the theory and mode of action of melatonin in AD will be discussed. Finally, the clinical studies supporting the therapeutic use of melatonin in AD will be critically assessed.

## 2. Basic Biology of Melatonin Relevant to Neurodegeneration

Circulating melatonin in mammals derives almost exclusively from the pineal gland [5]. In addition, melatonin is locally synthesized in many cells, tissues, and organs, including lymphocytes, bone marrow, the thymus, the gastrointestinal tract, skin, and the eyes (see [6,7]). The fact that the occurrence of melatonin is not restricted to vertebrates, but rather is almost ubiquitously present in numerous taxa including, e.g., bacteria, unicellular eukaryotes, and plants [6,8], underlines that this molecule has gained many additional functions in the course of evolution.

Both in other animals and in humans, melatonin participates in diverse physiological functions signaling not only the length of the night but also facilitating, among other, free radical scavenging and the immune response, thus, showing relevant cytoprotective properties [6]. Indeed, cytoprotection may well explain melatonin’s presence in most living organisms. The systemic and intracellular functions of melatonin differ in the amounts of agonist needed. Generally melatonin effects on biologic rhythms are exerted at nanomolar concentrations and via receptor-mediated mechanisms. The cytoprotective effects of melatonin needs 100–1000 higher concentrations, compatible with the amounts reached intracellularly by melatonin in several tissues [7].

Circulating melatonin binds to albumin [9] and is metabolized mainly in the liver primarily through hydroxylation at the C6 position. This is catalyzed selectively by the hepatic microsomal cytochrome P450 1A2 (CYP1A2), with minor contributions from hepatic CYP2C19 and the largely extrahepatic CYP1A1 and CYP1B11A [9,10,11,12]. The formed 6-hydroxymelatonin is then conjugated with sulphate or glucuronide to be excreted in the urine. Another important metabolite is *N*-acetylserotonin which is formed by *O*-demethylation, and may represent as much as 20% of a melatonin administered dose [13]. 

Melatonin is also metabolized in tissues by oxidative pyrrole ring cleavage into kynuramine derivatives. The primary cleavage product is *N*^1^-acetyl-*N*^2^-formyl-5-methoxykynuramine (AFMK), which is deformylated, either by arylamine formamidase or by hemoperoxidase, to *N*^1^-acetyl-5-methoxykynuramine (AMK) [14]. It has been proposed that AFMK is the primary active metabolite of melatonin to mediate cytoprotection [15]. Melatonin is also converted into cyclic 3-hydroxymelatonin in a process that directly scavenges two hydroxyl radicals [15]. Due to its rapid metabolism, melatonin has a short half-life of approximately one hour, although there is a marked inter-individual variation in plasma levels of melatonin after oral administration [16,17,18].

The two membrane melatonin receptors cloned so far (MT_1_ and MT_2_) have seven membrane domains and belong to the superfamily of G-protein coupled receptors [19]. MT_1_ and MT_2_ receptors are found in the cell membrane as dimers and heterodimers. GPR50, a G-protein coupled melatonin receptor ortholog that does not bind melatonin itself, dimerizes with MT_1_ receptors and can block melatonin binding [20]. The human MT_2_ receptor exhibits a lower affinity than the human MT_1_ receptor and becomes desensitized after exposure to melatonin, presumably by internalization.

As representatives of the G-protein coupled receptor family, MT_1_ and MT_2_ receptors act through a number of signal transduction mechanisms [19]. The MT_1_ receptor is coupled to G proteins that mediate adenylyl cyclase inhibition and phospholipase C activation. The MT_2_ receptor is also coupled to the inhibition of adenylyl cyclase, and it additionally inhibits the soluble guanylyl cyclase pathway.

By using receptor autoradiography with the nonselective 2-[125I]iodomelatonin ligand and real-time quantitative reverse transcription–polymerase chain reaction to label melatonin receptor mRNA, MT_1_ and MT_2_ receptors has been identified brain regions. At the level of the hippocampus, MT_2_ receptors were detected in CA3 and CA4 pyramidal neurons, which receive glutamatergic excitatory inputs from the entorhinal cortex, whereas MT_1_ receptors were predominantly expressed in CA1 [6].

Melatonin also binds to transcription factors belonging to the retinoic acid receptor superfamily, in particular, splice variants of RORα (RORα1, RORα2, and RORα isoform d) and RZRβ [21,22]. Retinoic acid receptor subforms are ubiquitously expressed in mammalian tissues [22].

Melatonin is a powerful antioxidant that scavenges ∙OH radicals as well as other radical oxygen species (ROS) and radical nitrogen species (RNS), and that gives rise to the cascade of metabolites mentioned above that share antioxidant properties [23]. Melatonin also acts indirectly to promote gene expression of antioxidant enzymes and to inhibit gene expression of prooxidant enzymes [24,25,26,27]. In particular, this holds for glutathione peroxidase (GPx) and for glutathione reductase (GRd), presumably in response to GPx-dependent increases in GSSG, the oxidized form of glutathione (GSH). Melatonin contributes to maintain normal brain GSH levels [28] by stimulating GSH biosynthesis via γ-glutamylcysteine synthase and glucose-6-phosphate dehydrogenase [26,29]. Melatonin has a demonstrated superiority to vitamin C and E in protection against oxidative damage and in scavenging free radicals [23].

Melatonin has significant anti-inflammatory properties presumably by inhibiting nuclear factor κB (NFκB) binding to DNA, thus, decreasing the synthesis of proinflammatory cytokines through inhibition of cyclooxygenase (Cox) [30], mainly Cox-2 [31], and by suppression of inducible nitric oxide (NO) synthase (iNOS) gene expression [32]. Melatonin was shown to protect from oxidative stress at physiological concentrations [23,33]. Although melatonin’s direct action as an antioxidant agent is mostly independent of receptor interaction [34], the upregulation of antioxidant enzymes by the methoxyindole involves nuclear transcription and in some cases RZR/RORα receptors [35].

The efficacy of melatonin in inhibiting oxidative damage has been tested in a variety of neurological disease models. In addition to the animals models of AD discussed below, melatonin has been shown to lower neural damage due to cadmium toxicity [36,37], hyperbaric hyperoxia [38,39], δ-aminolevulinic acid toxicity [40,41,42], γ radiation [43,44,45], focal ischemia [46,47], brain trauma [48,49,50], and a number of neurotoxins [51].

Melatonin’s neuroprotective properties, as well as its regulatory effects on circadian disturbances, validate its benefits as a therapeutic substance in the preventive treatment of AD. Moreover, melatonin exerts anti-excitatory, and at sufficient dosages, sedating effects [52,53], thus that a second neuroprotective mode of action may exist involving the γ-aminobutyric acid (GABA)-ergic system as a mediator. This view is supported by studies indicating that melatonin protects neurons from the toxicity of the amyloid-β (Aβ) peptide (a main neurotoxin involved in AD) via activation of GABA receptors [54].

Early studies on the anti-excitotoxic actions of melatonin employed kainate, an agonist of ionotropic glutamate receptors, and gave support to the hypothesis that melatonin prevents neuronal death induced by excitatory amino acids [55,56]. It has also been reported that administration of melatonin reduces the injury of hippocampal CA1 neurons caused by transient forebrain ischemia [57,58] or high glucocorticoid doses [59].

The various types of toxicities listed above can result in neuronal death by necrosis or apoptosis. Apoptotic neuronal death requires RNA and protein synthesis and depletion of trophic factors. Apoptosis also involves single-strand breaks of DNA and neurotrophic factors have been found to rescue neurons from this type of death [60]. They may act via cellular anti-apoptotic components, such as the B cell lymphoma proto-oncogene protein (Bcl-2). *In vitro* studies indicate that melatonin enhances expression of Bcl-2 and prevents apoptosis [61,62,63]. In addition, melatonin directly inhibits the opening of the mtPTP, thereby rescuing cells [64,65,66].

The aging process of the brain is presently known in sufficient detail [67]. A number of senescence processes rely on mitochondrial damage including apoptosis via cardiolipin peroxidation, cytochrome C release and mtPTP breakdown and reduction of the mitochondrial mass. The blockade of the electron transport chain leads to insufficient energy supply and impaired cell function and viability. The augmented ROS and RNS generation cause concomitant damage to endothelia, DNA proteins, and lipids. In addition neuronal overexcitation with calcium overload and activation of microglia occur with age. As a consequence of DNA damage, an increased telomere attrition takes place together with a reduction in cells with high proliferative capacity like immune progenitor cells thus leading to immunosenescence [67]. This situation is accompanied by the augmentation of autoimmune responses and by an inflammatory process leading to the increase of proinflammatory cytokines.

Evidence derived from animal studies indicates that melatonin may curtail most aspects of brain aging [67]. First, via action on central and peripheral circadian oscillators melatonin increases the depressed rhythm amplitudes and poor coordination of rhythms seen in the aged individuals. Melatonin affects clock protein expression and may modulate the disrupted metabolic sensing in senescence. Through the support of mitochondrial electron flux and reduction of electron overflow melatonin improves respiratory efficiency and energy supply and prevents apoptosis. The reduction of ROS and RNS exerted via radical scavenging, upregulation of antioxidant enzymes, inhibition of prooxidant enzymes, increase of GSH, and lower radical formation brings about a reduced damage to proteins, lipids, and DNA, together with inhibition of oxidant-induced telomere attrition and neuronal overexcitation. The antiinflammatory actions of melatonin are crucial in reducing aging-related processes, as well as the improved insulin sensitivity and counteraction of the metabolic syndrome attributed to the methoxyindole. Lastly, melatonin modulates natural and adaptive immunity and improves immunosenescence, in part via a greater number of cells with high proliferative capacity, including leukocytes, stem, and progenitor cells [67].

## 3. Overview of Melatonin Therapy for Alzheimer’s Disease—Theory and Mode of Action

Extracellular senile plaques, formed mainly by Aβ deposits, and intracellular neurofibrillary tangles, resulting mainly from abnormally hyperphosphorylated microtubule-associated protein (MAP) tau are the major pathological characteristic of AD. Aβ plays an important role in promoting neuronal degeneration in AD turning neurons vulnerable to age-related increases in the levels of oxidative stress and an altered cellular energy metabolism.

Aβ is composed by 39–43 amino acid residues derived from its precursor, the amyloid precursor protein (APP) [68]. APP is proteolytically processed by α- or β-secretases in different pathways. The α-non-amyloidogenic pathway involves cleavage of APP by α-secretase to release an *N*-terminal fragment of APP, which after cleavage by γ-secretase precludes the formation of the Aβ [68]. The β-amyloidogenic pathway includes β-secretase, which results in the formation of intact Aβ peptide and is mediated by the sequential cleavage of β-secretase and γ-secretase at the *N*- and *C*-terminal of Aβ sequence [68].

Melatonin inhibited the normal levels of soluble APP secretion in different cell lines interfering with APP maturation [69]. Additionally, the administration of melatonin efficiently reduces Aβ generation and deposition *in vivo* [70,71] and *in vitro* [69,72,73,74]. Generally, the results in transgenic mice support the view that melatonin should be given at an early phase to regulates APP and Aβ metabolism mainly by preventing their formation, with little anti-amyloid effect later on. Thus, melatonin therapy in old Tg2576 mice starting at 14 months of age could not prevent additional Aβ deposition [75] while a similar treatment starting at the fourth month of age was effective to reduce it [70]. As amyloid plaque pathology typically is seen in 10- to 12-month-old Tg2576 mice [76] the data point out to the effectiveness of melatonin in preventing early amyloid plaque formation rather than afterwards.

**Figure 1:**
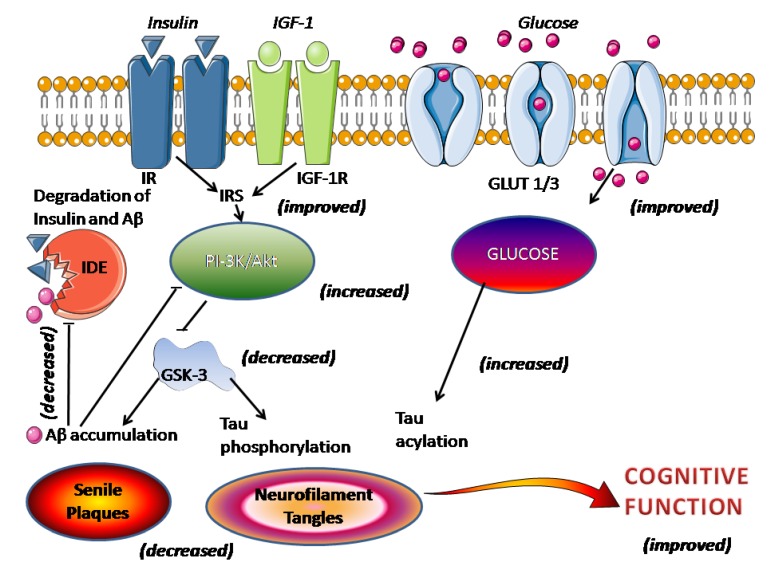
Effect of melatonin on impaired brain insulin signaling in AD. The figure schematizes the processes linking impaired insulin/insulin receptor with senile plaques and neurofibrillary tangles. In parentheses, the demonstrated action of melatonin as discussed in the text.

How melatonin exerts its inhibitory effect on the generation of Aβ remains undefined. The proteolytic cleavage of APP by α-secretase pathway is regulated by many physiological and pathological stimuli particularly through protein kinase (PK) C activation and secretase-mediated cleavage of APP. The inhibition of glycogen synthase kinase-3 (GSK-3) and upregulation of c-Jun *N*-terminal kinase result in high activity of matrix metalloproteinases with increasing degradation of Aβ [77]. The activity of insulin-degrading enzyme (IDE) that regulates the levels of insulin, Aβ and APP, decreased after Aβ increase [78]. GSK-3 interacts with presenilin-1, a cofactor of γ-secretase, the phosphorylation of GSK-3 by PKC leading to γ-secretase inactivation. Indeed, GSK-3 can be one of the common signaling pathways, increasing Aβ generation and tau hyperphosphorylation, and melatonin could regulate APP processing through PKC and GSK-3 pathways (Figure 1).

Melatonin interacts with Aβ_40_ and Aβ_42_ and inhibits progressive β-sheet and/or amyloid fibrils [79,80]. This interaction between melatonin and Aβ appears to depend on structural melatonin characteristics rather than on its antioxidant properties, as it could not be mimicked by other free radical scavengers [79]. By blocking the formation of secondary sheets, melatonin not only reduces neurotoxicity but also facilitates peptide clearance increasing proteolytic degradation, e.g., by IDE.

Oxidative stress plays a central role in Aβ-induced neurotoxicity and cell death. Accumulating data support that melatonin effectively protects cells against Aβ-induced oxidative damage and cell death *in vitro* [81,82] and *in vivo* [70,81,83,84,85]. In cells and animals treated with Aβ, melatonin could exert its protective activity through an antioxidant effect, whereas in APP transfected cells and transgenic animal models, the underlying mechanism may involve primarily the inhibition of generation of β-sheets and/or amyloid fibrils. Aggregated Aβ generates ROS that produce neuronal death through damage of neuronal membrane lipids, proteins and nucleic acids. Protection from Aβ toxicity by melatonin was observed, especially at the mitochondrial level [86,87].

The hyperphosphorylation of tau reduces tau capacity to prevent microtubule changes and the disruption of the cytoskeleton ensues [88,89]. The extent of neurofibrillary pathology correlates with the severity of dementia in AD patients. The level of hyperphosphorylated tau is three to four times higher in the brain of AD patients than in normal adult brains [90,91].

Melatonin efficiently attenuates tau hyperphosphorylation by affecting protein kinases and phosphatases in a number of experimental models including exposure of N2a and SH-SY5Y neuroblastoma cells to wortmannin [92], calyculin A [93,94,95], and okadaic acid [96,97,98,99]. Melatonin also antagonizes the oxidative stress given by these agents [100,101]. The inhibition of melatonin biosynthesis in rats not only resulted in impairment of spatial memory, but also induced an increase in tau phosphorylation, an effect prevented by melatonin supplementation [102].

Oxidative stress is known to influence tau phosphorylation state [103,104]. The accumulation of misfolded and aggregated proteins in brain neurons of AD is considered a consequence of oxidative stress, in addition to the molecular structural changes due to age [105]. As melatonin prevents, as an antioxidant and free radical scavenger, overproduction of free radicals, it seems feasible that the prevention of tau phosphorylation by melatonin is partly occurring due to its antioxidant activity. In addition several studies indicated melatonin may act as a modulator of enzymes in a way that is unrelated to its antioxidant properties. These include the regulation by melatonin of PKA [100,101], PKC [106,107], Ca^2+^/calmodulin-dependent kinase II [108,109,110] and mitogen-activated protein kinase [111].

An important factor in the pathogenesis of AD is the activation of microglia resulting in a higher level of expression of proinflammatory cytokines [112,113,114,115]. Epidemiological studies have shown that the use of anti-inflammatory drugs decreases the incidence of AD [116]. Aβ-induced microglial activation is a major source of inflammatory response [117]. Melatonin attenuated the production of proinflammatory cytokines induced by Aβ, NFκB, and NO in the rat brain [85,118]. Moreover, the DNA binding activity of NFκB was inhibited by melatonin [119,120].

Another major event in the pathogenesis of AD is the deficit in cholinergic function [121]. Neurons in the nucleus basalis of Meynert, the main source of cholinergic innervation to the cerebral cortex and the hippocampus, undergo a profound and selective degeneration in AD brains [122]. The levels of acetylcholine (ACh) are reduced at the early stage of AD whereas the activities of the synthesizing and degrading enzymes choline acetyltransferase (ChAT) and acetylcholinesterase (AChE) do not change until a late phase of AD [123,124]. As the decrease in ChAT activity in the neocortex of AD patients correlated with the severity of dementia, AChE inhibitors have become a standard treatment of mild to moderate AD [125].

Melatonin has a protective effect on the cholinergic system. It prevents the peroxynitrite-induced inhibition of choline transport and ChAT activity in synaptosomes and synaptic vesicles [126]. Melatonin treatment of eight-month-old APP695 transgenic mice significantly improved the profound reduction in ChAT activity in the frontal cortex and the hippocampus [81]. Melatonin also antagonizes the spatial memory deficit and the decreased ChAT activity found in adult ovariectomized rats [127]. However, in rats perfused intracerebroventricularly with Aβ for 14 days, melatonin was unable to restore ChAT activity [128]. Melatonin inhibited lipopolysaccharide- and streptozotocin-induced increase in AChE activity [129]. Recently hybrids of the AChE inhibitor tacrine and melatonin were synthesized as new drug candidates for treating AD [130,131]. These hybrids showed better antioxidant and cholinergic-preserving activity than tacrine or melatonin alone. The direct intracerebral administration of one of these hybrids decreased induced cell death and Aβ load in the APP/PS1 mouse brain parenchyma accompanied by a recovery of cognitive function [131].

There is a growing interest in the role of impaired brain insulin signaling in AD pathology. The disturbance of brain insulin/insulin-like growth factor 1 (IGF-1) signaling has been suggested to be a key causative event underlying AD, being linked to the presence of both senile plaques and neurofibrillary tangles [132,133,134]. This view, however, is not universally held [135]. An impaired insulin/insulin receptor (IR) signaling leads to decreased insulin-mediated activation of phosphoinositide 3-kinase (PI-3K)/Akt signaling activity, resulting in overactivation of GSK-3 which directly promotes tau hyperphosphorylation and Aβ accumulation and senile plaques formation (Figure 1). The activity of IDE, that regulates the levels of insulin, Aβ and APP, decreased after Aβ deposition [78]. The administration of melatonin reportedly reduces the signs of metabolic syndrome, such as hyperglycemia, dyslipidemia, hyperinsulinemia, insulin resistance, weight gain, and hypertension [136,137]. Melatonin restores insulin/insulin receptor mechanisms and increases phosphoinositide 3-kinase/Akt signaling activity with a resultant inhibition of GSK-3 and less Aβ accumulation and tau hyperphosphorylation (see [6]). Additionally, disruption of insulin signaling leads to a decreased glucose transporter-1 (GLUT-1) and -3 (GLUT-3) expression, culminating in impaired cerebral glucose uptake/metabolism, another event counteracted by melatonin. The restored neuronal glucose metabolism augments tau *N*-acetylglucosamine acylation, thus reducing tau hyperphosphorylation (Figure 1).

**Figure 2:**
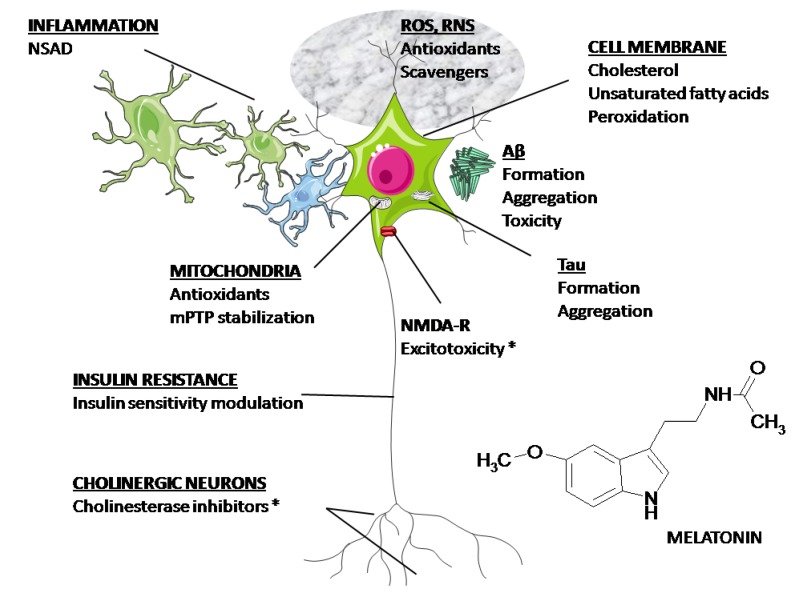
Possible targets for medication in AD. As discussed in the text, melatonin has the potential to affect all the mechanisms depicted in the figure. (*) Approved by the FDA.

Figure 2 summarizes the major possible targets for medication in AD. The Food and Drug Administration (FDA) has only approved AChE inhibitors and *N*-methyl-*D*-aspartate (NMDA) receptor blockers for clinical use. As discussed above, melatonin has the unique property to affect all the physiopathological mechanisms depicted in Figure 2. Hence, its potential as a neuroprotector in AD deserves to be explored.

## 4. Clinical Aspects of Melatonin Application in AD

In demented patients the severity of mental impairment correlated significantly with impaired nocturnal melatonin production [138]. This may be an early sign of the disease since cerebrospinal fluid (CSF) melatonin levels are reduced in AD patients autopsied at the preclinical stage Braak stage-1. Hence the reduction in CSF melatonin may be a marker for the early detection of AD [139].

Additionally plasma melatonin levels are lower in AD patients [140,141,142,143] and functionally impaired retinal-SCN-pineal connections were postulated as cause for this decrease [144]. Expression and activity of monoamine oxidase gene augmented, and β_1_-adrenoceptor mRNA levels decreased, in the pineal gland of AD patients [145]. In addition changes in melatonin receptor immunoreactivity occurred in the AD hippocampus [146,147]. Collectively the results indicate that replacement of melatonin levels can be a therapeutic strategy for arresting AD progression.

“Sundowning”, a chronobiological phenomenon that includes disorganized thinking, reduced ability to maintain attention to external stimuli, wandering, agitation, and perceptual and emotional disturbances, is a situation commonly observed in AD patients. As a time-of-day related phenomenon, appearing in late afternoon or early evening [148,149], the possible therapeutic effect of melatonin was entertained. Table 1 summarizes the data indicating that melatonin, as a chronobiotic agent, may be effective in treating sundowning and irregular sleep-wake cycles in AD patients.

A review of the published results concerning melatonin use in AD [150] yielded eight reports (five open-label studies, two case reports) (*N* = 89 patients) supporting a possible efficacy of melatonin: sleep quality improved and in patients with AD sundowning was reduced and cognitive decay slowed progression. In six double blind, randomized placebo-controlled trials, a total number of 210 AD patients were examined. Sleep quality increased and sundowning decreased significantly and cognitive performance improved in four studies (*N* = 143) whereas there was absence of effects in two studies (*N* = 67) [150].

Another systematic search of studies published between 1985 and April 2009 on melatonin and sundowning in AD patients was published [151]. All papers on melatonin treatment in dementia were retrieved and the effects of melatonin on circadian rhythm disturbances were scored by means of scoring sundowning/agitated behavior, sleep quality, and daytime functioning. A total of 9 papers, including 4 randomized controlled trials (*N* = 243), and 5 case series (*N* = 87) were reviewed (Table 1). Two of the randomized controlled trials found a significant improvement in sundowning/agitated behavior. All five case series found an improvement [151]. Thus, whether melatonin has any value in treating AD remains uncertain. Probably, the heterogeneity of the group examined in fully developed AD makes it difficult to disclose any therapeutic effect in studies including a small number of patients [152]. Moreover, the reduced hippocampal expression of MT_2_ melatonin receptors in AD patients [146] and of MT_1_ receptors in the circadian apparatus may explain why melatonin treatment is erratic at this advanced stage of the disease [153,154].

**Table 1:** Studies including treatment of Alzheimer’s disease (AD) patients with melatonin.

Design	Subjects	Treatment	Time	Measured	Results	Reference
Open-label study	10 AD patients	3 mg melatonin p.o./daily at bed time	3 weeks	Daily logs of sleep and wake quality completed by caretakers	7 out of 10 dementia patients having sleep disorders treated with melatonin showed a significant decrease in sundowning and reduced variability of sleep onset time	[155]
Open-label study	14 AD patients	9 mg melatonin p.o./daily at bed time	22 to 35 months	Daily logs of sleep and wake quality completed by caretakers. Neuro-psychological assessment	Sundowning was no longer detectable in 12 patients and persisted, although attenuated in 2 patients. A significant improvement of sleep quality was found. Lack of progression of the cognitive and behavioral signs of the disease during the time they received melatonin	[156]
Case report	Mono-zygotic twins with AD of 8 years duration	One of the patients was treated with melatonin 9 mg p.o./daily at bed time.	36 months	Neuro-psychological assessment. Neuroimaging	Sleep and cognitive function severely impaired in the twin not receiving melatonin as compared to the melatonin-treated twin	[157]
Open-label study	11 AD patients	3 mg melatonin p.o./daily at bed time	3 weeks	Daily logs of sleep and wake quality	Significant decrease in agitated behaviors in all three shifts; significant decrease in daytime sleepiness	[158]
Open-label, placebo-controlled trial	14 AD patients	6 mg melatonin p.o./daily at bed time or placebo	4 weeks	Daily logs of sleep and wake quality completed by caretakers. Actigraphy	AD patients receiving melatonin showed a significantly reduced percentage of nighttime activity compared to a placebo group	[159]
Randomized double blind placebo-controlled cross over study	25 AD patients	6 mg of slow release melatonin p.o. or placebo at bed time	7 weeks	Actigraphy	Melatonin had no effect on median total time asleep, number of awakenings or sleep efficiency	[160]
Open-label study	45 AD patients	6–9 mg melatonin p.o./daily at bed time	4 months	Daily logs of sleep and wake quality completed by caretakers. Neuro-psychological assessment	Melatonin improved sleep and suppressed sundowning, an effect seen regardless of the concomitant medication employed	[161]
Randomized placebo-controlled clinical trial	157 AD patients	2.5-mg slow-release melatonin, or 10-mg melatonin or placebo at bed time	2 months	Actigraphy. Caregiver ratings of sleep quality	Non significant trends for increased nocturnal total sleep time and decreased wake after sleep onset in the melatonin groups. Caregiver ratings of sleep quality showed a significant improvement in the 2.5-mg sustained-release melatonin group relative to placebo	[162]
Double-blind, placebo-controlled study	20 AD patients	Placebo or 3 mg melatonin p.o./daily at bed time	4 weeks	Actigraphy. Neuro-psychological assessment	Melatonin significantly prolonged the sleep time and decreased activity in the night. Cognitive function was improved by melatonin	[163]
Open-label study	7 AD patients	3 mg melatonin p.o./daily at bed time	3 weeks	Actigraphy. Neuro-psychological assessment.	Complete remission of day-night rhythm disturbances or sundowning was seen in 4 patients, with partial remission in other 2	[164]
Randomized placebo-controlled study	17 AD patients	3 mg melatonin p.o./daily at bed time (7 patients). Placebo (10 patients)	2 weeks	Actigraphy. Neuro-psychological assessment.	In melatonin-treated group, actigraphic nocturnal activity and agitation showed significant reductions compared to baseline	[165]
Case report	68-year-old man with AD who developed rapid eye movement (REM) sleep behavior disorder	5–10 mg melatonin p.o./daily at bed time.	20 months	Polysomno-graphy	Melatonin was effective to suppress REM sleep behavior disorder	[166]
Randomized placebo-controlled study	50 AD patients	Morning light exposure (2500 lux, 1 h) and 5 mg melatonin (*N* = 16) or placebo (*N* = 17) in the evening	10 weeks	Actigraphy	Light treatment alone did not improve nighttime sleep, daytime wake, or rest-activity rhythm. Light treatment plus melatonin increased daytime wake time and activity levels and strengthened the rest-activity rhythm	[167]
Randomized placebo-controlled study	41 AD patients	Melatonin (8.5 mg immediate release and 1.5 mg sustained release) (*N* = 24) or placebo (*N* = 17) administered at 22:00 h	10 days	Actigraphy	There were no significant effects of melatonin, compared with placebo, on sleep, circadian rhythms, or agitation	[168]

p.o.: *per os*.

Mild cognitive impairment (MCI) is the name given to signs and symptoms diagnosed in those who have an objective and measurable deficit in cognitive functions, but with a relative preservation of daily activities. Annual conversion rates to AD are as high as 10%–15% [169] and MCI represents a clinically important stage for identifying and treating individuals at risk. Indeed, the degenerative process in AD brain starts 20–30 years before the clinical onset of AD [170,171]. During this phase, the loads of plaques and tangles increase and at a certain threshold the first symptoms appear [172,173]. As mentioned above, CSF melatonin levels decrease in preclinical stages of AD, suggesting that the reduction of CSF melatonin may be an early trigger and marker for AD [139,145]. Although it is not known whether the relative melatonin deficiency is either a consequence or a cause of neurodegeneration, the loss in melatonin presumably aggravates the disease.

We previously reported a retrospective analysis in which daily 3–9 mg of a fast-release melatonin preparation *per os* (p.o.) at bedtime for up to three years significantly improved cognitive and emotional performance and daily sleep/wake cycle in 25 MCI patients [174]. Recently we reported data from another series of 96 MCI outpatients, 61 of who had received daily 3–24 mg of a fast-release melatonin preparation p.o. at bedtime for 15 to 60 months in comparison to a similar group of 35 MCI patients who did not receive it [175]. In addition, all patients received the individual standard medication considered appropriate by the attending psychiatrist.

Patients treated with melatonin exhibited significantly better performance in Mini–Mental State Examination and the cognitive subscale of the AD Assessment Scale. After application of a neuropsychological battery comprising a Mattis’ test, Digit-symbol test, Trail A and B tasks and the Rey’s verbal test, better performance was found in melatonin-treated patients for every parameter tested [175]. Abnormally high Beck Depression Inventory scores decreased in melatonin-treated patients, concomitantly with the improvement in the quality of sleep and wakefulness [175]. These results further support that melatonin is a useful add-on drug for treating MCI in a clinical environment.

Thus, an early initiation of melatonin treatment can be decisive for therapeutic success [75]. In Table 2, published data concerning melatonin treatment in MCI are summarized. Six double blind, randomized placebo-controlled trials and two open-label retrospective studies (*N* = 782) consistently showed that the administration of daily evening melatonin improves sleep quality and cognitive performance in MCI patients. Therefore, melatonin treatment could be effective at early stages of the neurodegenerative disease.

There are two reasons why it is beneficial to use melatonin in MCI patients. In the course of the neurodegenerative process, the age-related deterioration in circadian organization becomes significantly exacerbated and is responsible of behavioral problems like sundowning [176]. Age-related cognitive decline in healthy older adults can be predicted by the fragmentation of the circadian rhythm in locomotor behavior. Hence, replacement of the low melatonin levels occurring in brain [139,145] can be highly beneficial in MCI patients. 

On the other hand, the bulk of information on the neuroprotective properties of melatonin derived from experimental studies (see for [177,178]) makes it highly desirable to employ pharmacological doses in MCI patients with the aim of arresting or slowing disease progression. Although the pineal gland secretes melatonin that circulates in the blood and the CSF [179], recent data support the hypothesis that, in sheep, and presumably in humans, only CSF melatonin, and not bloodstream melatonin, can provide most of melatonin to the cerebral tissue in high concentrations [180]. Significant concentration gradients oriented from the ventricle (close to the CSF) to the cerebral tissue, with concentrations varying by a factor of 1 to 125 were demonstrated. Hence, besides supporting the role of CSF in physiological availability of melatonin [179] these results imply that high, pharmacological amounts of melatonin must be given to have access to the brain.

The mechanisms accounting for the therapeutic effect of melatonin in MCI patients remain to be defined. Melatonin treatment mainly promotes slow wave sleep in the elderly [181] and can be beneficial in MCI by augmenting the restorative phases of sleep, including the augmented secretion of GH and neurotrophins.

As outlined above, melatonin acts at different levels relevant to the development and manifestation of AD. The antioxidant, mitochondrial, and antiamyloidogenic effects can possibly interfere with the onset of the disease. Therefore, the time when melatonin treatment begins can be decisive for the final response [75].

One important aspect to be considered is the melatonin dose employed, which may be unnecessarily low when one takes into consideration the binding affinities, half-life, and relative potencies of the different melatonin agonists on the market. In addition to being generally more potent than the native molecule, melatonin analogs are employed in considerably higher amounts [182]. Licensed doses of the melatonin receptor agonist ramelteon vary from 8 to 32 mg/day while agomelatine has been licensed for treatment of major depressive disorder at doses of 25–50 mg/day. In clinical studies involving healthy human subjects, tasimelteon (Vanda Pharmaceuticals, Washington, DC, USA), another melatonin receptor agonist recently approved by the FDA is administered at doses of 20 to 100 mg/day [183] while pharmacokinetics, pharmacodynamics and safety of the melatonin receptor agonist TIK-301 (Tikvah Pharmaceuticals, Atlanta, GA, USA) have been examined in a placebo controlled study using 20 to 100 mg/day [184]. Therefore, studies of MCI with melatonin doses in the range of 100–300 mg/day are further warranted.

A combination therapy of melatonin with a melatonin receptor agonist could theoretically be beneficial for AD patients, especially if the melatonin receptor agonist has a better pharmacokinetic profile than melatonin. However, melatonin receptor agonists have shown limited benefit in murine models of AD [185].

As melatonin is cleared very rapidly from the bloodstream strategies such as inhibition of melatonin degradation and clearance from the body could be a useful add-on to exogenous melatonin treatment. Indeed, pharmacological plasma levels of melatonin could be affected as a result of concurrent exposure to chemicals that modulate the expression of CYP1A2. For example, fluvoxamine, a potent inhibitor of CYP1A2 and to a lesser extent of CYP2C19, augmented plasma melatonin levels [186,187]. Likewise, the concomitant consumption of caffeine whose metabolism is principally catalyzed by CYP1A2, more than doubled plasma levels and increased the bioavailability of melatonin [188]. Another candidate is 5-methoxypsoralen, a drug used for the treatment of psoriasis, which elevates plasma levels of endogenous and exogenous melatonin [189,190,191].

It should be stressed that melatonin has a high safety profile, it is usually remarkably well tolerated and, in some studies, it has been administered to patients at very large doses [16,192,193,194,195]. Melatonin (300 mg/day) for up to three years decreased oxidative stress in patients with amyotrophic lateral sclerosis [192]. 

In children with muscular dystrophy, 70 mg/day of melatonin reduced cytokines and lipid peroxidation [193]. Doses of 80 mg melatonin hourly for 4 h were given to healthy men with no undesirable effects other than drowsiness [16]. In healthy women, given 300 mg melatonin/day for four months, there were no side effects [194]. A recent randomized controlled double-blind clinical trial on 50 patients referred for liver surgery indicated that a single preoperative enteral dose of 50 mg/kg melatonin (*i.e.*, an equivalent to 3 g for a 60-kg adult) was safe and well tolerated [195].

Another outcome of the study recently reported [175] was that when melatonin is employed much less benzodiazepines are needed to treat sleep disturbances in MCI. Since, as above-mentioned, melatonin and benzodiazepines shared some neurochemical (*i.e.*, interaction with GABA-mediated mechanisms in brain [196]) and behavioral properties (e.g., a similar day-dependent anxiolytic activity [46]) melatonin therapy was postulated to be an effective tool to decrease the dose of benzodiazepines needed in patients [155,197,198,199]. A recent retrospective analysis of a German prescriptions database identified 512 patients who had initiated treatment with prolonged release melatonin (2 mg) over a 10-month period [200]. From 112 patients in this group who had previously used benzodiazepines, 31% discontinued treatment with benzodiazepines three months after beginning prolonged release melatonin treatment. The discontinuation rate was higher in patients receiving two or three melatonin prescription [200]. The prolonged use of benzodiazepines and benzodiazepine receptor agonists (*Z*-drugs) is related to severe withdrawal symptoms and potential dependency, which has become a public health issue leading to multiple campaigns to decrease consumption of these drugs. A recent pharmacoepidemiological study concluded that these campaigns generally failed when they were not associated with the availability and market of melatonin [201]

## 5. Conclusions

In conclusion, the question as to whether melatonin has a therapeutic value in preventing or treating MCI, affecting disease initiation or progression of the neuropathology and the mechanisms involved, deserves to be further analyzed. Double-blind multicenter studies are needed to explore and investigate the potential and usefulness of melatonin as an antidementia drug at the early stage of disease.

As melatonin exhibits both hypnotic and chronobiotic properties, it has been therapeutically used for treatment of age-related insomnia as well as of other primary and secondary insomnia [202,203]. A consensus of the British Association for Psychopharmacology on evidence-based treatment of insomnia, parasomnia, and circadian rhythm sleep disorders concluded that melatonin at a dose of 2 mg is the first choice treatment when a hypnotic is indicated in patients over 55 years [204].

As shown by the binding affinities, half-life and relative potencies of the different melatonin agonists in the market it is clear that studies using these low doses of melatonin are unsuitable to give appropriate comparison with the effect of the above mentioned compounds, which in addition to being generally more potent than the native molecule are employed in considerably higher amounts [205]. Therefore, further studies employing higher melatonin doses are needed to clarify its potential therapeutical neuroprotective implications in humans. From animal studies it is clear that a number of preventive effects of melatonin, such as those in neurodegenerative disorders, need high doses of melatonin to become apparent [177]. If one expects melatonin to be an effective neuroprotector it is likely that the low doses of melatonin employed thus far are not very beneficial.

**Table 2:** Studies including treatment of mild cognitive impairment (MCI) patients with melatonin.

Design	Subjects	Treatment	Time	Measured	Results	Reference
Double-blind, placebo-controlled, crossover study	10 patients with MCI	6 mg melatonin p.o./daily at bed time	10 days	Actigraphy. Neuro-psychological assessment	Melatonin enhanced the rest-activity rhythm and improved sleep quality. The ability to remember previously learned items improved along with a significant reduction in depressed mood	[206]
Double-blind, placebo-controlled pilot study	26 patients with age-related MCI	1 mg melatonin p.o. or placebo at bed time	4 weeks	Sleep questionnaire and cognitive tests at baseline and at 4 weeks	Melatonin administration improved reported morning “restedness” and sleep latency after nocturnal awakening. It also improved scores on the California Verbal Learning Test-interference subtest.	[207]
Randomizeddouble blind, placebo-controlled study	354 patients with age-related MCI	Prolonged release melatonin (Circadin, 2 mg) or placebo, 2 h before bedtime	3 weeks	Leeds Sleep Evaluation and Pittsburgh Sleep QuestionnairesClinical Global Improvement scale score and quality of life.	Melatonin resulted in significant and clinically meaningful improvements in sleep quality, morning alertness, sleep onset latency and quality of life	[208]
Open-label, retrospective study	60 MCI out-patients	35 patients received daily 3–9 mg of a fast-release melatonin preparation p.o. at bedtime. Melatonin was given in addition to the standard medication	9–24 months	Daily logs of sleep and wake quality. Initial and final neuro-psychological assessment.	Abnormally high Beck Depression Inventory scores decreased in melatonin-treated patients, concomitantly with an improvement in wakefulness and sleep quality. Patients treated with melatonin showed significantly better performance in neuropsychological assessment.	[174]
Long-term, double-blind, placebo-controlled, 2 × 2 factorial randomized study	189 patients with age-related cognitive decay	Long-term daily treatment with whole-day bright (1000 lux) or dim (300 lux) light. Evenin*g* melatonin (2.5 mg) or placebo	1 to 3.5 years	Standardized scales for cognitive and noncognitive symptoms, limitations of activities of daily living, and adverse effects assessed every 6 months.	Light attenuated cognitive deterioration and ameliorated depressive symptoms. Melatonin shortened sleep onset latency and increased sleep duration but adversely affected scores for depression. The combined treatment of bright light plus melatonin showed the best effects.	[209]
Prospective, randomized, double-blind, placebo-controlled, study	22 patients with age-related cognitive decay	Patients received 2 months of melatonin (5 mg p.o./day) and 2 months of placebo	2 months	Sleep disorders were evaluated with the Northside Hospital Sleep Medicine Institute test. Behavioral disorders were evaluated with the Yesavage Geriatric Depression Scale and Goldberg Anxiety Scale.	Melatonin treatment significantly improved sleep quality scores. Depression also improved significantly after melatonin administration.	[210]
Randomizeddouble-blind, placebo-controlled study	25 MCI out-patients	11 patients received an oily emulsion of docosa-hexaenoic acid-phospho-lipids containing melatonin (10 mg) and tryptophan (190 mg)	12 weeks	Neuro-psychological assessment of orientation and cognitive functions, short-term and long-term memory, attentional abilities, executive functions, visuo-constructional and visuo-spatial abilities, language and mood.	Older adults with MCI had significant improvements in several measures of cognitive function when supplemented with the oily emulsion containing melatonin and tryptophan for 12 weeks, compared with the placebo. The antioxidant capacity of erythrocytes and membrane lipid composition improved after treatment.	[211,212]
Open-label, retrospective study	96 MCI out-patients	61 patients received daily 3–24 mg of a fast-release melatonin preparation p.o. at bedtime. Melatonin was given in addition to the standard medication	15–60 months	Daily logs of sleep and wake quality. Initial and final neuro-psychological assessment.	Abnormally high Beck Depression Inventory scores decreased in melatonin-treated patients, concomitantly with an improvement in wakefulness and sleep quality. Patients treated with melatonin showed significantly better performance in neuropsychological assessment. Only 6 out of 61 patients treated with melatonin needed concomitant benzodiazepine treatment *vs.* 22 out of 35 MCI patients not receiving melatonin.	[175]

## Abbreviations

Ach: acetylcholine
AChE: acetylcholinesterase
AD: Alzheimer’s disease
ADI: Alzheimer’s Disease International
AFMK: *N*^1^-acetyl-*N*^2^-formyl-5-methoxykynuramine
Akt: protein kinase identified in the AKT virus
AMK: *N*^1^-acetyl-5-methoxykynuramine
APP: amyloid precursor protein
Aβ: aggregated β-amyloid
Bcl-2: B cell lymphoma proto-oncogene protein
ChAT: choline acetyltransferase
Cox: cyclooxygenase
CSF: cerebrospinal fluid
CYP1A1: cytochrome P450 1A1
CYP1A2: cytochrome P450 1A2
CYP2C19: cytochrome P450 2C19
CYP1B1: cytochrome P450 1B1
GABA: γ-aminobutyric acid
GPR50: G-protein receptor 50 ortholog
GLUT-1: glucose transporter-1
GLUT-3: glucose transporter-3
GPx: glutathione peroxidase
GRd: glutathione reductase
GSH: reduced glutathione
GSK-3: glycogen synthase kinase-3
IDE: insulin-degrading enzyme
iNOS: inducible nitric oxide synthase
IGF-1: Insulin-like growth factor 1
MAP: microtubule-associated protein
MCI: mild cognitive impairment
mPTP: mitochondrial permeability transition pore
mRNA: messenger ribonucleic acid
MT_1_: melatonin receptor 1
MT_2_: melatonin receptor 2
NFκB: nuclear factor κB
NMDA: *N*-methyl-d-aspartate
nNOS: neuronal nitric oxide synthase
NO: nitric oxide
NSAD: non-steroidal anti-inflammatory drugs
PI3-K: phosphoinositide 3-kinase
PK: protein kinase
REM: rapid eye movement
RNS: reactive nitrogen species
ROR: retinoic acid receptor-related orphan receptor
ROS: reactive oxygen species
RZR: retinoid Z receptor
SCN: suprachiasmatic nuclei
SOD: superoxide dismutase

## References

[1] Reiter R.J., Garcia J.J., Pie J. (1998). Oxidative toxicity in models of neurodegeneration: Responses to melatonin. Restor. Neurol. Neurosci..

[2] ADI G8 Policy Briefing Reveals 135 Million People will Live with Dementia by 2050. http://www.alz.co.uk/news/g8-policy-brief-reveals-135-million-people-with-dementia-by-2050.

[3] Johnson E.J., Vishwanathan R., Johnson M.A., Hausman D.B., Davey A., Scott T.M., Green R.C., Miller L.S., Gearing M., Woodard J. (2013). Relationship between serum and brain carotenoids, α-tocopherol, and retinol concentrations and cognitive performance in the oldest old from the Georgia Centenarian Study. J. Aging Res..

[4] Bubenik G.A., Konturek S.J. (2011). Melatonin and aging: Prospects for human treatment. J. Physiol Pharmacol..

[5] Claustrat B., Brun J., Chazot G. (2005). The basic physiology and pathophysiology of melatonin. Sleep Med. Rev..

[6] Hardeland R., Cardinali D.P., Srinivasan V., Spence D.W., Brown G.M., Pandi-Perumal S.R. (2011). Melatonin—A pleiotropic, orchestrating regulator molecule. Prog. Neurobiol..

[7] Venegas C., Garcia J.A., Escames G., Ortiz F., Lopez A., Doerrier C., Garcia-Corzo L., Lopez L.C., Reiter R.J., Acuña-Castroviejo D. (2012). Extrapineal melatonin: Analysis of its subcellular distribution and daily fluctuations. J. Pineal Res..

[8] Paredes S.D., Korkmaz A., Manchester L.C., Tan D.X., Reiter R.J. (2009). Phytomelatonin: A review. J. Exp. Bot..

[9] Cardinali D.P., Lynch H.J., Wurtman R.J. (1972). Binding of melatonin to human and rat plasma proteins. Endocrinology.

[10] Ma X., Idle J.R., Krausz K.W., Gonzalez F.J. (2005). Metabolism of melatonin by human cytochromes p450. Drug Metab. Dispos..

[11] Facciola G., Hidestrand M., von Bahr C., Tybring G. (2001). Cytochrome P450 isoforms involved in melatonin metabolism in human liver microsomes. Eur. J. Clin. Pharmacol..

[12] Skene D.J., Papagiannidou E., Hashemi E., Snelling J., Lewis D.F., Fernandez M., Ioannides C. (2001). Contribution of CYP1A2 in the hepatic metabolism of melatonin: Studies with isolated microsomal preparations and liver slices. J. Pineal Res..

[13] Young I.M., Leone R.M., Francis P., Stovell P., Silman R.E. (1985). Melatonin is metabolized to *N*-acetyl serotonin and 6-hydroxymelatonin in man. J. Clin. Endocrinol. Metab..

[14] Hardeland R., Tan D.X., Reiter R.J. (2009). Kynuramines, metabolites of melatonin and other indoles: The resurrection of an almost forgotten class of biogenic amines. J. Pineal Res..

[15] Tan D.X., Manchester L.C., Terron M.P., Flores L.J., Reiter R.J. (2007). One molecule, many derivatives: A never-ending interaction of melatonin with reactive oxygen and nitrogen species?. J. Pineal Res..

[16] Waldhauser F., Waldhauser M., Lieberman H.R., Deng M.H., Lynch H.J., Wurtman R.J. (1984). Bioavailability of oral melatonin in humans. Neuroendocrinology.

[17] Aldhous M., Franey C., Wright J., Arendt J. (1985). Plasma concentrations of melatonin in man following oral absorption of different preparations. Br. J. Clin. Pharmacol..

[18] Fourtillan J.B., Brisson A.M., Gobin P., Ingrand I., Decourt J.P., Girault J. (2000). Bioavailability of melatonin in humans after day-time administration of D_7_ melatonin. Biopharm. Drug Dispos..

[19] Dubocovich M.L., Delagrange P., Krause D.N., Sugden D., Cardinali D.P., Olcese J. (2010). International Union of Basic and Clinical Pharmacology. LXXV. Nomenclature, classification, and pharmacology of G protein-coupled melatonin receptors. Pharmacol. Rev..

[20] Levoye A., Dam J., Ayoub M.A., Guillaume J.L., Couturier C., Delagrange P., Jockers R. (2006). The orphan GPR_50_ receptor specifically inhibits MT_1_ melatonin receptor function through heterodimerization. EMBO J..

[21] Wiesenberg I., Missbach M., Kahlen J.P., Schrader M., Carlberg C. (1995). Transcriptional activation of the nuclear receptor RZR alpha by the pineal gland hormone melatonin and identification of CGP 52608 as a synthetic ligand. Nucleic Acids Res..

[22] Lardone P.J., Guerrero J.M., Fernandez-Santos J.M., Rubio A., Martin-Lacave I., Carrillo-Vico A. (2011). Melatonin synthesized by T lymphocytes as a ligand of the retinoic acid-related orphan receptor. J. Pineal Res..

[23] Galano A., Tan D.X., Reiter R.J. (2011). Melatonin as a natural ally against oxidative stress: A physicochemical examination. J. Pineal Res..

[24] Antolin I., Rodriguez C., Sainz R.M., Mayo J.C., Uria H., Kotler M.L., Rodriguez-Colunga M.J., Tolivia D., Menendez-Pelaez A. (1996). Neurohormone melatonin prevents cell damage: Effect on gene expression for antioxidant enzymes. FASEB J..

[25] Pablos M.I., Reiter R.J., Ortiz G.G., Guerrero J.M., Agapito M.T., Chuang J.I., Sewerynek E. (1998). Rhythms of glutathione peroxidase and glutathione reductase in brain of chick and their inhibition by light. Neurochem. Int..

[26] Rodriguez C., Mayo J.C., Sainz R.M., Antolin I., Herrera F., Martin V., Reiter R.J. (2004). Regulation of antioxidant enzymes: A significant role for melatonin. J. Pineal Res..

[27] Jimenez-Ortega V., Cano P., Cardinali D.P., Esquifino A.I. (2009). 24-Hour variation in gene expression of redox pathway enzymes in rat hypothalamus: Effect of melatonin treatment. Redox Rep..

[28] Subramanian P., Mirunalini S., Pandi-Perumal S.R., Trakht I., Cardinali D.P. (2007). Melatonin treatment improves the antioxidant status and decreases lipid content in brain and liver of rats. Eur. J. Pharmacol..

[29] Kilanczyk E., Bryszewska M. (2003). The effect of melatonin on antioxidant enzymes in human diabetic skin fibroblasts. Cell. Mol. Biol. Lett..

[30] Cardinali D.P., Ritta M.N., Fuentes A.M., Gimeno M.F., Gimeno A.L. (1980). Prostaglandin E release by rat medial basal hypothalamus *in vitro*. Inhibition by melatonin at submicromolar concentrations. Eur. J. Pharmacol..

[31] Deng W.G., Tang S.T., Tseng H.P., Wu K.K. (2006). Melatonin suppresses macrophage cyclooxygenase-2 and inducible nitric oxide synthase expression by inhibiting p52 acetylation and binding. Blood.

[32] Costantino G., Cuzzocrea S., Mazzon E., Caputi A.P. (1998). Protective effects of melatonin in zymosan-activated plasma-induced paw inflammation. Eur. J. Pharmacol..

[33] Tan D., Reiter R.J., Chen L.D., Poeggeler B., Manchester L.C., Barlow-Walden L.R. (1994). Both physiological and pharmacological levels of melatonin reduce DNA adduct formation induced by the carcinogen safrole. Carcinogenesis.

[34] Leon-Blanco M.M., Guerrero J.M., Reiter R.J., Pozo D. (2004). RNA expression of human telomerase subunits TR and TERT is differentially affected by melatonin receptor agonists in the MCF-7 tumor cell line. Cancer Lett..

[35] Urata Y., Honma S., Goto S., Todoroki S., Iida T., Cho S., Honma K., Kondo T. (1999). Melatonin induces γ-glutamylcysteine synthetase mediated by activator protein-1 in human vascular endothelial cells. Free Radic. Biol. Med..

[36] Poliandri A.H., Esquifino A.I., Cano P., Jimenez V., Lafuente A., Cardinali D.P., Duvilanski B.H. (2006). *In vivo* protective effect of melatonin on cadmium-induced changes in redox balance and gene expression in rat hypothalamus and anterior pituitary. J. Pineal Res..

[37] Jimenez-Ortega V., Cano P., Scacchi P.A., Cardinali D.P., Esquifino A.I. (2011). Cadmium-induced disruption in 24-h expression of clock and redox enzyme genes in rat medial basal hypothalamus. Prevention by melatonin. Front. Neurol..

[38] Shaikh A.Y., Xu J., Wu Y., He L., Hsu C.Y. (1997). Melatonin protects bovine cerebral endothelial cells from hyperoxia-induced DNA damage and death. Neurosci. Lett..

[39] Pablos M.I., Reiter R.J., Chuang J.I., Ortiz G.G., Guerrero J.M., Sewerynek E., Agapito M.T., Melchiorri D., Lawrence R., Deneke S.M. (1997). Acutely administered melatonin reduces oxidative damage in lung and brain induced by hyperbaric oxygen. J. Appl. Physiol..

[40] Princ F.G., Juknat A.A., Maxit A.G., Cardalda C., Batlle A. (1997). Melatonin’s antioxidant protection against δ-aminolevulinic acid-induced oxidative damage in rat cerebellum. J. Pineal Res..

[41] Carneiro R.C., Reiter R.J. (1998). δ-Aminolevulinic acid-induced lipid peroxidation in rat kidney and liver is attenuated by melatonin: An *in vitro* and *in vivo* study. J. Pineal Res..

[42] Onuki J., Almeida E.A., Medeiros M.H., Di M.P. (2005). Inhibition of 5-aminolevulinic acid-induced DNA damage by melatonin, *N*^1^-acetyl-*N*^2^-formyl-5-methoxykynuramine, quercetin or resveratrol. J. Pineal Res..

[43] Erol F.S., Topsakal C., Ozveren M.F., Kaplan M., Ilhan N., Ozercan I.H., Yildiz O.G. (2004). Protective effects of melatonin and vitamin E in brain damage due to gamma radiation: An experimental study. Neurosurg. Rev..

[44] Shirazi A., Haddadi G.H., Asadi-Amoli F., Sakhaee S., Ghazi-Khansari M., Avand A. (2011). Radioprotective effect of melatonin in reducing oxidative stress in rat lenses. Cell J..

[45] Taysi S., Memisogullari R., Koc M., Yazici A.T., Aslankurt M., Gumustekin K., Al B., Ozabacigil F., Yilmaz A., Tahsin O.H. (2008). Melatonin reduces oxidative stress in the rat lens due to radiation-induced oxidative injury. Int. J. Radiat. Biol..

[46] Lee E.J., Wu T.S., Lee M.Y., Chen T.Y., Tsai Y.Y., Chuang J.I., Chang G.L. (2004). Delayed treatment with melatonin enhances electrophysiological recovery following transient focal cerebral ischemia in rats. J. Pineal Res..

[47] Tai S.H., Hung Y.C., Lee E.J., Lee A.C., Chen T.Y., Shen C.C., Chen H.Y., Lee M.Y., Huang S.Y., Wu T.S. (2011). Melatonin protects against transient focal cerebral ischemia in both reproductively active and estrogen-deficient female rats: The impact of circulating estrogen on its hormetic dose-response. J. Pineal Res..

[48] Beni S.M., Kohen R., Reiter R.J., Tan D.X., Shohami E. (2004). Melatonin-induced neuroprotection after closed head injury is associated with increased brain antioxidants and attenuated late-phase activation of NF-κB and AP-1. FASEB J..

[49] Tsai M.C., Chen W.J., Tsai M.S., Ching C.H., Chuang J.I. (2011). Melatonin attenuates brain contusion-induced oxidative insult, inactivation of signal transducers and activators of transcription 1, and upregulation of suppressor of cytokine signaling-3 in rats. J. Pineal Res..

[50] Kabadi S.V., Maher T.J. (2010). Posttreatment with uridine and melatonin following traumatic brain injury reduces edema in various brain regions in rats. Ann. N. Y. Acad. Sci..

[51] Reiter R.J., Manchester L.C., Tan D.X. (2010). Neurotoxins: Free radical mechanisms and melatonin protection. Curr. Neuropharmacol..

[52] Golombek D.A., Pevet P., Cardinali D.P. (1996). Melatonin effects on behavior: Possible mediation by the central GABAergic system. Neurosci. Biobehav. Rev..

[53] Caumo W., Levandovski R., Hidalgo M.P. (2009). Preoperative anxiolytic effect of melatonin and clonidine on postoperative pain and morphine consumption in patients undergoing abdominal hysterectomy: A double-blind, randomized, placebo-controlled study. J. Pain.

[54] Louzada P.R., Paula Lima A.C., Mendonca-Silva D.L., Noel F., de Mello F.G., Ferreira S.T. (2004). Taurine prevents the neurotoxicity of beta-amyloid and glutamate receptor agonists: Activation of GABA receptors and possible implications for Alzheimer’s disease and other neurological disorders. FASEB J..

[55] Giusti P., Lipartiti M., Franceschini D., Schiavo N., Floreani M., Manev H. (1996). Neuroprotection by melatonin from kainate-induced excitotoxicity in rats. FASEB J..

[56] Manev H., Uz T., Kharlamov A., Cagnoli C.M., Franceschini D., Giusti P. (1996). *In vivo* protection against kainate-induced apoptosis by the pineal hormone melatonin: Effect of exogenous melatonin and circadian rhythm. Restor. Neurol. Neurosci..

[57] Cho S., Joh T.H., Baik H.H., Dibinis C., Volpe B.T. (1997). Melatonin administration protects CA1 hippocampal neurons after transient forebrain ischemia in rats. Brain Res..

[58] Kilic E., Ozdemir Y.G., Bolay H., Kelestimur H., Dalkara T. (1999). Pinealectomy aggravates and melatonin administration attenuates brain damage in focal ischemia. J. Cereb. Blood Flow Metab..

[59] Furio A.M., Fontao R., Falco N., Ruiz J.I., Caccuri R.L., Cardinali D.P. (2008). Neuroprotective effect of melatonin on glucocorticoid toxicity in the rat hippocampus. Open Physiol. J..

[60] Dodd S., Maes M., Anderson G., Dean O.M., Moylan S., Berk M. (2013). Putative neuroprotective agents in neuropsychiatric disorders. Prog. Neuropsychopharmacol. Biol. Psychiatry.

[61] Jiao S., Wu M.M., Hu C.L., Zhang Z.H., Mei Y.A. (2004). Melatonin receptor agonist 2-iodomelatonin prevents apoptosis of cerebellar granule neurons via K^+^ current inhibition. J. Pineal Res..

[62] Koh P.O. (2011). Melatonin prevents down-regulation of astrocytic phosphoprotein PEA-15 in ischemic brain injury. J. Pineal Res..

[63] Radogna F., Diederich M., Ghibelli L. (2010). Melatonin: A pleiotropic molecule regulating inflammation. Biochem. Pharmacol..

[64] Peng T.I., Hsiao C.W., Reiter R.J., Tanaka M., Lai Y.K., Jou M.J. (2012). mtDNA T8993G mutation-induced mitochondrial complex V inhibition augments cardiolipin-dependent alterations in mitochondrial dynamics during oxidative, Ca^2+^, and lipid insults in NARP cybrids: A potential therapeutic target for melatonin. J. Pineal Res..

[65] Jou M.J. (2011). Melatonin preserves the transient mitochondrial permeability transition for protection during mitochondrial Ca^2+^ stress in astrocyte. J. Pineal Res..

[66] Andrabi S.A., Sayeed I., Siemen D., Wolf G., Horn T.F. (2004). Direct inhibition of the mitochondrial permeability transition pore: A possible mechanism responsible for anti-apoptotic effects of melatonin. FASEB J..

[67] Hardeland R. (2013). Melatonin and the theories of aging: A critical appraisal of melatonin’s role in antiaging mechanisms. J. Pineal Res..

[68] Selkoe D.J. (2004). Cell biology of protein misfolding: The examples of Alzheimer’s and Parkinson’s diseases. Nat. Cell Biol..

[69] Lahiri D.K., Ghosh C. (1999). Interactions between melatonin, reactive oxygen species, and nitric oxide. Ann. N. Y. Acad. Sci..

[70] Matsubara E., Bryant-Thomas T., Pacheco Q.J., Henry T.L., Poeggeler B., Herbert D., Cruz-Sanchez F., Chyan Y.J., Smith M.A., Perry G. (2003). Melatonin increases survival and inhibits oxidative and amyloid pathology in a transgenic model of Alzheimer’s disease. J. Neurochem..

[71] Lahiri D.K., Chen D., Ge Y.W., Bondy S.C., Sharman E.H. (2004). Dietary supplementation with melatonin reduces levels of amyloid β-peptides in the murine cerebral cortex. J. Pineal Res..

[72] Song W., Lahiri D.K. (1997). Melatonin alters the metabolism of the beta-amyloid precursor protein in the neuroendocrine cell line PC12. J. Mol. Neurosci..

[73] Zhang Y.C., Wang Z.F., Wang Q., Wang Y.P., Wang J.Z. (2004). Melatonin attenuates β-amyloid-induced inhibition of neurofilament expression. Acta Pharmacol. Sin..

[74] Olivieri G., Hess C., Savaskan E., Ly C., Meier F., Baysang G., Brockhaus M., Muller-Spahn F. (2001). Melatonin protects SHSY5Y neuroblastoma cells from cobalt-induced oxidative stress, neurotoxicity and increased β-amyloid secretion. J. Pineal Res..

[75] Quinn J., Kulhanek D., Nowlin J., Jones R., Pratico D., Rokach J., Stackman R. (2005). Chronic melatonin therapy fails to alter amyloid burden or oxidative damage in old Tg2576 mice: Implications for clinical trials. Brain Res..

[76] Hsiao K., Chapman P., Nilsen S., Eckman C., Harigaya Y., Younkin S., Yang F., Cole G. (1996). Correlative memory deficits, Aβ elevation, and amyloid plaques in transgenic mice. Science.

[77] Donnelly P.S., Caragounis A., Du T., Laughton K.M., Volitakis I., Cherny R.A., Sharples R.A., Hill A.F., Li Q.X., Masters C.L. (2008). Selective intracellular release of copper and zinc ions from bis (thiosemicarbazonato) complexes reduces levels of Alzheimer disease amyloid-β peptide. J. Biol. Chem..

[78] Farris W., Mansourian S., Chang Y., Lindsley L., Eckman E.A., Frosch M.P., Eckman C.B., Tanzi R.E., Selkoe D.J., Guenette S. (2003). Insulin-degrading enzyme regulates the levels of insulin, amyloid β-protein, and the β-amyloid precursor protein intracellular domain *in vivo*. Proc. Natl. Acad. Sci. USA.

[79] Poeggeler B., Miravalle L., Zagorski M.G., Wisniewski T., Chyan Y.J., Zhang Y., Shao H., Bryant-Thomas T., Vidal R., Frangione B. (2001). Melatonin reverses the profibrillogenic activity of apolipoprotein E4 on the Alzheimer amyloid Aβ peptide. Biochemistry.

[80] Pappolla M., Bozner P., Soto C., Shao H., Robakis N.K., Zagorski M., Frangione B., Ghiso J. (1998). Inhibition of Alzheimer β-fibrillogenesis by melatonin. J. Biol. Chem..

[81] Feng Z., Chang Y., Cheng Y., Zhang B.L., Qu Z.W., Qin C., Zhang J.T. (2004). Melatonin alleviates behavioral deficits associated with apoptosis and cholinergic system dysfunction in the APP 695 transgenic mouse model of Alzheimer’s disease. J. Pineal Res..

[82] Zatta P., Tognon G., Carampin P. (2003). Melatonin prevents free radical formation due to the interaction between β-amyloid peptides and metal ions [Al^III^, Zn^II^, Cu^II^, Mn^II^, Fe^II^]. J. Pineal Res..

[83] Furio A.M., Cutrera R.A., Castillo Thea V., Perez L.S., Riccio P., Caccuri R.L., Brusco L.L., Cardinali D.P. (2002). Effect of melatonin on changes in locomotor activity rhythm of Syrian hamsters injected with β amyloid peptide 25–35 in the suprachiasmatic nuclei. Cell. Mol. Neurobiol..

[84] Shen Y.X., Xu S.Y., Wei W., Sun X.X., Yang J., Liu L.H., Dong C. (2002). Melatonin reduces memory changes and neural oxidative damage in mice treated with d-galactose. J. Pineal Res..

[85] Rosales-Corral S., Tan D.X., Reiter R.J., Valdivia-Velazquez M., Martinez-Barboza G., Acosta-Martinez J.P., Ortiz G.G. (2003). Orally administered melatonin reduces oxidative stress and proinflammatory cytokines induced by amyloid-β peptide in rat brain: A comparative, *in vivo* study *versus* vitamin C and, E. J. Pineal Res..

[86] Olcese J.M., Cao C., Mori T., Mamcarz M.B., Maxwell A., Runfeldt M.J., Wang L., Zhang C., Lin X., Zhang G. (2009). Protection against cognitive deficits and markers of neurodegeneration by long-term oral administration of melatonin in a transgenic model of Alzheimer disease. J. Pineal Res..

[87] Dragicevic N., Copes N., O’Neal-Moffitt G., Jin J., Buzzeo R., Mamcarz M., Tan J., Cao C., Olcese J.M., Arendash G.W. (2011). Melatonin treatment restores mitochondrial function in Alzheimer’s mice: A mitochondrial protective role of melatonin membrane receptor signaling. J. Pineal Res..

[88] Brion J.P., Anderton B.H., Authelet M., Dayanandan R., Leroy K., Lovestone S., Octave J.N., Pradier L., Touchet N., Tremp G. (2001). Neurofibrillary tangles and tau phosphorylation. Biochem. Soc. Symp..

[89] Billingsley M.L., Kincaid R.L. (1997). Regulated phosphorylation and dephosphorylation of tau protein: Effects on microtubule interaction, intracellular trafficking and neurodegeneration. Biochem. J..

[90] Khatoon S., Grundke-Iqbal I., Iqbal K. (1992). Brain levels of microtubule-associated protein tau are elevated in Alzheimer’s disease: A radioimmuno-slot-blot assay for nanograms of the protein. J. Neurochem..

[91] Iqbal K., Alonso A.C., Chen S., Chohan M.O., El-Akkad E., Gong C.X., Khatoon S., Li B., Liu F., Rahman A. (2005). Tau pathology in Alzheimer disease and other tauopathies. Biochim. Biophys. Acta.

[92] Deng Y.Q., Xu G.G., Duan P., Zhang Q., Wang J.Z. (2005). Effects of melatonin on wortmannin-induced tau hyperphosphorylation. Acta Pharmacol. Sin..

[93] Li S.P., Deng Y.Q., Wang X.C., Wang Y.P., Wang J.Z. (2004). Melatonin protects SH-SY5Y neuroblastoma cells from calyculin A-induced neurofilament impairment and neurotoxicity. J. Pineal Res..

[94] Li X.C., Wang Z.F., Zhang J.X., Wang Q., Wang J.Z. (2005). Effect of melatonin on calyculin A-induced tau hyperphosphorylation. Eur. J. Pharmacol..

[95] Xiong Y.F., Chen Q., Chen J., Zhou J., Wang H.X. (2011). Melatonin reduces the impairment of axonal transport and axonopathy induced by calyculin A. J. Pineal Res..

[96] Benitez-King G., Tunez I., Bellon A., Ortiz G.G., Anton-Tay F. (2003). Melatonin prevents cytoskeletal alterations and oxidative stress induced by okadaic acid in N1E-115 cells. Exp. Neurol..

[97] Montilla-Lopez P., Munoz-Agueda M.C., Feijoo L.M., Munoz-Castaneda J.R., Bujalance-Arenas I., Tunez-Finana I. (2002). Comparison of melatonin *versus* vitamin C on oxidative stress and antioxidant enzyme activity in Alzheimer’s disease induced by okadaic acid in neuroblastoma cells. Eur. J. Pharmacol..

[98] Montilla P., Feijoo M., Munoz M.C., Munoz-Castaneda J.R., Bujalance I., Tunez I. (2003). Effect of melatonin on the oxidative stress in N1E-115 cells is not mediated by MT1 receptors. J. Physiol. Biochem..

[99] Wang Y.P., Li X.T., Liu S.J., Zhou X.W., Wang X.C., Wang J.Z. (2004). Melatonin ameliorated okadaic-acid induced Alzheimer-like lesions. Acta Pharmacol. Sin..

[100] Liu S.J., Wang J.Z. (2002). Alzheimer-like tau phosphorylation induced by wortmannin *in vivo* and its attenuation by melatonin. Acta Pharmacol. Sin..

[101] Wang X.C., Zhang J., Yu X., Han L., Zhou Z.T., Zhang Y., Wang J.Z. (2005). Prevention of isoproterenol-induced tau hyperphosphorylation by melatonin in the rat. Sheng Li Xue Bao.

[102] Zhu L.Q., Wang S.H., Ling Z.Q., Wang D.L., Wang J.Z. (2004). Effect of inhibiting melatonin biosynthesis on spatial memory retention and tau phosphorylation in rat. J. Pineal Res..

[103] Gomez-Ramos A., Diaz-Nido J., Smith M.A., Perry G., Avila J. (2003). Effect of the lipid peroxidation product acrolein on tau phosphorylation in neural cells. J. Neurosci. Res..

[104] Lovell M.A., Xiong S., Xie C., Davies P., Markesbery W.R. (2004). Induction of hyperphosphorylated tau in primary rat cortical neuron cultures mediated by oxidative stress and glycogen synthase kinase-3. J. Alzheimers Dis..

[105] Kenyon C.J. (2010). The genetics of ageing. Nature.

[106] Schuster C., Williams L.M., Morris A., Morgan P.J., Barrett P. (2005). The human MT_1_ melatonin receptor stimulates cAMP production in the human neuroblastoma cell line SH-SY5Y cells via a calcium-calmodulin signal transduction pathway. J. Neuroendocrinol..

[107] Peschke E., Muhlbauer E., Musshoff U., Csernus V.J., Chankiewitz E., Peschke D. (2002). Receptor (MT_1_) mediated influence of melatonin on cAMP concentration and insulin secretion of rat insulinoma cells INS-1. J. Pineal Res..

[108] Witt-Enderby P.A., MacKenzie R.S., McKeon R.M., Carroll E.A., Bordt S.L., Melan M.A. (2000). Melatonin induction of filamentous structures in non-neuronal cells that is dependent on expression of the human MT_1_ melatonin receptor. Cell Motil. Cytoskelet..

[109] Rivera-Bermudez M.A., Gerdin M.J., Earnest D.J., Dubocovich M.L. (2003). Regulation of basal rhythmicity in protein kinase C activity by melatonin in immortalized rat suprachiasmatic nucleus cells. Neurosci. Lett..

[110] Benitez-King G., Rios A., Martinez A., Anton-Tay F. (1996). *In vitro* inhibition of Ca^2+^/calmodulin-dependent kinase II activity by melatonin. Biochim. Biophys. Acta.

[111] Chan A.S., Lai F.P., Lo R.K., Voyno-Yasenetskaya T.A., Stanbridge E.J., Wong Y.H. (2002). Melatonin MT_1_ and MT_2_ receptors stimulate c-Jun *N*-terminal kinase via pertussis toxin-sensitive and -insensitive G proteins. Cell Signal..

[112] Arends Y.M., Duyckaerts C., Rozemuller J.M., Eikelenboom P., Hauw J.J. (2000). Microglia, amyloid and dementia in alzheimer disease. A correlative study. Neurobiol. Aging.

[113] Combadiere C., Feumi C., Raoul W., Keller N., Rodero M., Pezard A., Lavalette S., Houssier M., Jonet L., Picard E. (2007). CX3CR1-dependent subretinal microglia cell accumulation is associated with cardinal features of age-related macular degeneration. J. Clin. Investig..

[114] Streit W.J., Mrak R.E., Griffin W.S. (2004). Microglia and neuroinflammation: A pathological perspective. J. Neuroinflamm..

[115] Shen Y., Zhang G., Liu L., Xu S. (2007). Suppressive effects of melatonin on amyloid-β-induced glial activation in rat hippocampus. Arch. Med. Res..

[116] Stuchbury G., Munch G. (2005). Alzheimer’s associated inflammation, potential drug targets and future therapies. J. Neural Transm..

[117] Park S.Y., Jin M.L., Kim Y.H., Kim Y., Lee S.J. (2012). Anti-inflammatory effects of aromatic-turmerone through blocking of NF-κB, JNK, and p38 MAPK signaling pathways in amyloid beta-stimulated microglia. Int. Immunopharmacol..

[118] Lau W.W., Ng J.K., Lee M.M., Chan A.S., Wong Y.H. (2012). Interleukin-6 autocrine signaling mediates melatonin MT_1/2_ receptor-induced STAT_3_ Tyr_705_ phosphorylation. J. Pineal Res..

[119] Mohan N., Sadeghi K., Reiter R.J., Meltz M.L. (1995). The neurohormone melatonin inhibits cytokine, mitogen and ionizing radiation induced NF-κB. Biochem. Mol. Biol. Int..

[120] Chuang J.I., Mohan N., Meltz M.L., Reiter R.J. (1996). Effect of melatonin on NF-κB DNA-binding activity in the rat spleen. Cell Biol. Int..

[121] Struble R.G., Cork L.C., Whitehouse P.J., Price D.L. (1982). Cholinergic innervation in neuritic plaques. Science.

[122] Samuel W., Masliah E., Hill L.R., Butters N., Terry R. (1994). Hippocampal connectivity and Alzheimer’s dementia: Effects of synapse loss and tangle frequency in a two-component model. Neurology.

[123] Terry A.V., Buccafusco J.J. (2003). The cholinergic hypothesis of age and Alzheimer’s disease-related cognitive deficits: Recent challenges and their implications for novel drug development. J. Pharmacol. Exp. Ther..

[124] Rinne J.O., Laine M., Hiltunen J., Erkinjuntti T. (2003). Semantic decision making in early probable AD: A PET activation study. Brain Res. Cogn. Brain Res..

[125] Spencer J.P., Middleton L.J., Davies C.H. (2010). Investigation into the efficacy of the acetylcholinesterase inhibitor, donepezil, and novel procognitive agents to induce γ oscillations in rat hippocampal slices. Neuropharmacology.

[126] Guermonprez L., Ducrocq C., Gaudry-Talarmain Y.M. (2001). Inhibition of acetylcholine synthesis and tyrosine nitration induced by peroxynitrite are differentially prevented by antioxidants. Mol. Pharmacol..

[127] Feng Z., Cheng Y., Zhang J.T. (2004). Long-term effects of melatonin or 17 β-estradiol on improving spatial memory performance in cognitively impaired, ovariectomized adult rats. J. Pineal Res..

[128] Tang F., Nag S., Shiu S.Y., Pang S.F. (2002). The effects of melatonin and Ginkgo biloba extract on memory loss and choline acetyltransferase activities in the brain of rats infused intracerebroventricularly with β-amyloid 1-40. Life Sci..

[129] Agrawal R., Tyagi E., Shukla R., Nath C. (2009). A study of brain insulin receptors, AChE activity and oxidative stress in rat model of ICV STZ induced dementia. Neuropharmacology.

[130] Fernandez-Bachiller M.I., Perez C., Campillo N.E., Paez J.A., Gonzalez-Munoz G.C., Usan P., Garcia-Palomero E., Lopez M.G., Villarroya M., Garcia A.G. (2009). Tacrine-melatonin hybrids as multifunctional agents for Alzheimer’s disease, with cholinergic, antioxidant, and neuroprotective properties. ChemMedChem.

[131] Spuch C., Antequera D., Isabel Fernandez-Bachiller M., Isabel Rodriguez-Franco M., Carro E. (2010). A new tacrine-melatonin hybrid reduces amyloid burden and behavioral deficits in a mouse model of Alzheimer’s disease. Neurotox. Res..

[132] O’Neill C., Kiely A.P., Coakley M.F., Manning S., Long-Smith C.M. (2012). Insulin and IGF-1 signalling: Longevity, protein homoeostasis and Alzheimer’s disease. Biochem. Soc. Trans..

[133] Hildreth K.L., van Pelt R.E., Schwartz R.S. (2012). Obesity, insulin resistance, and Alzheimer’s disease. Obesity.

[134] De la Monte S.M. (2012). Brain insulin resistance and deficiency as therapeutic targets in Alzheimer’s disease. Curr. Alzheimer Res..

[135] Thambisetty M., Jeffrey M.E., Yang A., Dolan H., Marano C., Zonderman A.B., Troncoso J.C., Zhou Y., Wong D.F., Ferrucci L. (2013). Glucose intolerance, insulin resistance, and pathological features of Alzheimer disease in the Baltimore Longitudinal Study of Aging. JAMA Neurol..

[136] Cardinali D.P., Cano P., Jimenez-Ortega V., Esquifino A.I. (2011). Melatonin and the metabolic syndrome: Physiopathologic and therapeutical implications. Neuroendocrinology.

[137] Cardinali D.P., Bernasconi P.A., Reynoso R., Toso C.F., Scacchi P. (2013). Melatonin may curtail the metabolic syndrome: Studies on initial and fully established fructose-induced metabolic syndrome in rats. Int. J. Mol. Sci..

[138] Magri F., Locatelli M., Balza G., Molla G., Cuzzoni G., Fioravanti M., Solerte S.B., Ferrari E. (1997). Changes in endocrine circadian rhythms as markers of physiological and pathological brain aging. Chronobiol. Int..

[139] Zhou J.N., Liu R.Y., Kamphorst W., Hofman M.A., Swaab D.F. (2003). Early neuropathological Alzheimer’s changes in aged individuals are accompanied by decreased cerebrospinal fluid melatonin levels. J. Pineal Res..

[140] Skene D.J., Vivien-Roels B., Sparks D.L., Hunsaker J.C., Pevet P., Ravid D., Swaab D.F. (1990). Daily variation in the concentration of melatonin and 5-methoxytryptophol in the human pineal gland: Effect of age and Alzheimer’s disease. Brain Res..

[141] Ohashi Y., Okamoto N., Uchida K., Iyo M., Mori N., Morita Y. (1999). Daily rhythm of serum melatonin levels and effect of light exposure in patients with dementia of the Alzheimer’s type. Biol. Psychiatry.

[142] Liu R.Y., Zhou J.N., van Heerikhuize J., Hofman M.A., Swaab D.F. (1999). Decreased melatonin levels in postmortem cerebrospinal fluid in relation to aging, Alzheimer’s disease, and apolipoprotein E-epsilon4/4 genotype. J. Clin. Endocrinol. Metab..

[143] Mishima K., Tozawa T., Satoh K., Matsumoto Y., Hishikawa Y., Okawa M. (1999). Melatonin secretion rhythm disorders in patients with senile dementia of Alzheimer’s type with disturbed sleep-waking. Biol. Psychiatry.

[144] Skene D.J., Swaab D.F. (2003). Melatonin rhythmicity: Effect of age and Alzheimer’s disease. Exp. Gerontol..

[145] Wu Y.H., Feenstra M.G., Zhou J.N., Liu R.Y., Torano J.S., van Kan H.J., Fischer D.F., Ravid R., Swaab D.F. (2003). Molecular changes underlying reduced pineal melatonin levels in Alzheimer disease: Alterations in preclinical and clinical stages. J. Clin. Endocrinol. Metab..

[146] Savaskan E., Ayoub M.A., Ravid R., Angeloni D., Fraschini F., Meier F., Eckert A., Muller-Spahn F., Jockers R. (2005). Reduced hippocampal MT_2_ melatonin receptor expression in Alzheimer’s disease. J. Pineal Res..

[147] Savaskan E., Olivieri G., Meier F., Brydon L., Jockers R., Ravid R., Wirz-Justice A., Muller-Spahn F. (2002). Increased melatonin 1a-receptor immunoreactivity in the hippocampus of Alzheimer’s disease patients. J. Pineal Res..

[148] Weldemichael D.A., Grossberg G.T. (2010). Circadian rhythm disturbances in patients with Alzheimer’s disease: A review. Int. J. Alzheimers Dis..

[149] Klaffke S., Staedt J. (2006). Sundowning and circadian rhythm disorders in dementia. Acta Neurol. Belg..

[150] Cardinali D.P., Furio A.M., Brusco L.I. (2010). Clinical aspects of melatonin intervention in Alzheimer’s disease progression. Curr. Neuropharmacol..

[151] De Jonghe A., Korevaar J.C., van Munster B.C., de Rooij S.E. (2010). Effectiveness of melatonin treatment on circadian rhythm disturbances in dementia. Are there implications for delirium? A systematic review. Int. J. Geriatr. Psychiatry.

[152] Pappolla M.A., Chyan Y.J., Poeggeler B., Frangione B., Wilson G., Ghiso J., Reiter R.J. (2000). An assessment of the antioxidant and the antiamyloidogenic properties of melatonin: Implications for Alzheimer’s disease. J. Neural Transm..

[153] Wu Y.H., Swaab D.F. (2005). The human pineal gland and melatonin in aging and Alzheimer’s disease. J. Pineal Res..

[154] Wu Y.H., Zhou J.N., van Heerikhuize J., Jockers R., Swaab D.F. (2007). Decreased MT_1_ melatonin receptor expression in the suprachiasmatic nucleus in aging and Alzheimer’s disease. Neurobiol. Aging.

[155] Fainstein I., Bonetto A., Brusco L.I., Cardinali D.P. (1997). Effects of melatonin in elderly patients with sleep disturbance. A pilot study. Curr. Ther. Res..

[156] Brusco L.I., Marquez M., Cardinali D.P. (1998). Melatonin treatment stabilizes chronobiologic and cognitive symptoms in Alzheimer’s disease. Neuro Endocrinol. Lett..

[157] Brusco L.I., Marquez M., Cardinali D.P. (1998). Monozygotic twins with Alzheimer’s disease treated with melatonin: Case report. J. Pineal Res..

[158] Cohen-Mansfield J., Garfinkel D., Lipson S. (2000). Melatonin for treatment of sundowning in elderly persons with dementia—A preliminary study. Arch. Gerontol. Geriatr..

[159] Mishima K., Okawa M., Hozumi S., Hishikawa Y. (2000). Supplementary administration of artificial bright light and melatonin as potent treatment for disorganized circadian rest-activity and dysfunctional autonomic and neuroendocrine systems in institutionalized demented elderly persons. Chronobiol. Int..

[160] Serfaty M., Kennell-Webb S., Warner J., Blizard R., Raven P. (2002). Double blind randomised placebo controlled trial of low dose melatonin for sleep disorders in dementia. Int. J. Geriatr. Psychiatry.

[161] Cardinali D.P., Brusco L.I., Liberczuk C., Furio A.M. (2002). The use of melatonin in Alzheimer’s disease. Neuro Endocrinol. Lett..

[162] Singer C., Tractenberg R.E., Kaye J., Schafer K., Gamst A., Grundman M., Thomas R., Thal L.J. (2003). A multicenter, placebo-controlled trial of melatonin for sleep disturbance in Alzheimer’s disease. Sleep.

[163] Asayama K., Yamadera H., Ito T., Suzuki H., Kudo Y., Endo S. (2003). Double blind study of melatonin effects on the sleep-wake rhythm, cognitive and non-cognitive functions in Alzheimer type dementia. J. Nippon Med. Sch..

[164] Mahlberg R., Kunz D., Sutej I., Kuhl K.P., Hellweg R. (2004). Melatonin treatment of day-night rhythm disturbances and sundowning in Alzheimer disease: An open-label pilot study using actigraphy. J. Clin. Psychopharmacol..

[165] Mahlberg R., Walther S. (2007). Actigraphy in agitated patients with dementia. Monitoring treatment outcomes. Z. Gerontol. Geriatr..

[166] Anderson K.N., Jamieson S., Graham A.J., Shneerson J.M. (2008). REM sleep behaviour disorder treated with melatonin in a patient with Alzheimer’s disease. Clin. Neurol. Neurosurg..

[167] Dowling G.A., Burr R.L., van Someren E.J., Hubbard E.M., Luxenberg J.S., Mastick J., Cooper B.A. (2008). Melatonin and bright-light treatment for rest-activity disruption in institutionalized patients with Alzheimer’s disease. J. Am. Geriatr. Soc..

[168] Gehrman P.R., Connor D.J., Martin J.L., Shochat T., Corey-Bloom J., Ancoli-Israel S. (2009). Melatonin fails to improve sleep or agitation in double-blind randomized placebo-controlled trial of institutionalized patients with Alzheimer disease. Am. J. Geriatr. Psychiatry.

[169] Farias S.T., Mungas D., Reed B.R., Harvey D., DeCarli C. (2009). Progression of mild cognitive impairment to dementia in clinic- *vs* community-based cohorts. Arch. Neurol..

[170] Davies L., Wolska B., Hilbich C., Multhaup G., Martins R., Simms G., Beyreuther K., Masters C.L. (1988). A_4_ amyloid protein deposition and the diagnosis of Alzheimer’s disease: Prevalence in aged brains determined by immunocytochemistry compared with conventional neuropathologic techniques. Neurology.

[171] Price J.L., Morris J.C. (1999). Tangles and plaques in nondemented aging and “preclinical” Alzheimer’s disease. Ann. Neurol..

[172] Braak H., Braak E. (1995). Staging of Alzheimer’s disease-related neurofibrillary changes. Neurobiol. Aging.

[173] Braak H., Braak E. (1998). Evolution of neuronal changes in the course of Alzheimer’s disease. J. Neural Transm. Suppl..

[174] Furio A.M., Brusco L.I., Cardinali D.P. (2007). Possible therapeutic value of melatonin in mild cognitive impairment. A retrospective study. J. Pineal Res..

[175] Cardinali D.P., Vigo D.E., Olivar N., Vidal M.F., Furio A.M., Brusco L.I. (2012). Therapeutic application of melatonin in mild cognitive impairment. Am. J. Neurodegener Dis..

[176] Wu Y.H., Swaab D.F. (2007). Disturbance and strategies for reactivation of the circadian rhythm system in aging and Alzheimer’s disease. Sleep Med..

[177] Pandi-Perumal S.R., BaHammam A.S., Brown G.M., Spence D.W., Bharti V.K., Kaur C., Hardeland R., Cardinali D.P. (2013). Melatonin antioxidative defense: Therapeutical implications for aging and neurodegenerative processes. Neurotox. Res..

[178] Rosales-Corral S.A., Acuña-Castroviejo D., Coto-Montes A., Boga J.A., Manchester L.C., Fuentes-Broto L., Korkmaz A., Ma S., Tan D.X., Reiter R.J. (2012). Alzheimer’s disease: Pathological mechanisms and the beneficial role of melatonin. J. Pineal Res..

[179] Tan D.X., Manchester L.C., Sanchez-Barcelo E., Mediavilla M.D., Reiter R.J. (2010). Significance of high levels of endogenous melatonin in mammalian cerebrospinal fluid and in the central nervous system. Curr. Neuropharmacol..

[180] Legros C., Chesneau D., Boutin J.A., Barc C., Malpaux B. (2014). Melatonin from cerebrospinal fluid, but not from blood, reaches sheep cerebral tissues under physiological conditions. J. Neuroendocrinol..

[181] Monti J.M., Alvarino F., Cardinali D.P., Savio I., Pintos A. (1999). Polysomnographic study of the effect of melatonin on sleep in elderly patients with chronic primary insomnia. Arch. Gerontol. Geriatr..

[182] Cardinali D.P., Srinivasan V., Brzezinski A., Brown G.M. (2012). Melatonin and its analogs in insomnia and depression. J. Pineal Res..

[183] Rajaratnam S.M., Polymeropoulos M.H., Fisher D.M., Roth T., Scott C., Birznieks G., Klerman E.B. (2009). Melatonin agonist tasimelteon (VEC-162) for transient insomnia after sleep-time shift: Two randomised controlled multicentre trials. Lancet.

[184] Mulchahey J.J., Goldwater D.R., Zemlan F.P. (2004). A single blind, placebo controlled, across groups dose escalation study of the safety, tolerability, pharmacokinetics and pharmacodynamics of the melatonin analog β-methyl-6-chloromelatonin. Life Sci..

[185] McKenna J.T., Christie M.A., Jeffrey B.A., McCoy J.G., Lee E., Connolly N.P., Ward C.P., Strecker R.E. (2012). Chronic ramelteon treatment in a mouse model of Alzheimer’s disease. Arch. Ital. Biol..

[186] Ursing C., Hartter S., von Bahr C., Tybring G., Bertilsson L., Rojdmark S. (2002). Does hepatic metabolism of melatonin affect the endogenous serum melatonin level in man?. J. Endocrinol. Investig..

[187] Hartter S., Grozinger M., Weigmann H., Roschke J., Hiemke C. (2000). Increased bioavailability of oral melatonin after fluvoxamine coadministration. Clin. Pharmacol. Ther..

[188] Hartter S., Nordmark A., Rose D.M., Bertilsson L., Tybring G., Laine K. (2003). Effects of caffeine intake on the pharmacokinetics of melatonin, a probe drug for CYP1A2 activity. Br. J. Clin. Pharmacol..

[189] Souetre E., Salvati E., Belugou J.L., de Galeani B., Krebs B., Ortonne J.P., Darcourt G. (1987). 5-Methoxypsoralen increases the plasma melatonin levels in humans. J. Investig. Dermatol..

[190] Garde E., Micic S., Knudsen K., Angelo H.R., Wulf H.C. (1994). 8-Methoxypsoralen increases daytime plasma melatonin levels in humans through inhibition of metabolism. Photochem. Photobiol..

[191] Mauviard F., Raynaud F., Geoffriau M., Claustrat B., Pevet P. (1995). 5-Methoxypsoralen inhibits 6-hydroxylation of melatonin in the rat. Biol. Signals.

[192] Weishaupt J.H., Bartels C., Polking E., Dietrich J., Rohde G., Poeggeler B., Mertens N., Sperling S., Bohn M., Huther G. (2006). Reduced oxidative damage in ALS by high-dose enteral melatonin treatment. J. Pineal Res..

[193] Chahbouni M., Escames G., Venegas C., Sevilla B., Garcia J.A., Lopez L.C., Munoz-Hoyos A., Molina-Carballo A., Acuña-Castroviejo D. (2010). Melatonin treatment normalizes plasma pro-inflammatory cytokines and nitrosative/oxidative stress in patients suffering from Duchenne muscular dystrophy. J. Pineal Res..

[194] Voordouw B.C., Euser R., Verdonk R.E., Alberda B.T., de Jong F.H., Drogendijk A.C., Fauser B.C., Cohen M. (1992). Melatonin and melatonin-progestin combinations alter pituitary-ovarian function in women and can inhibit ovulation. J. Clin. Endocrinol. Metab..

[195] Nickkholgh A., Schneider H., Sobirey M., Venetz W.P., Hinz U., Pelzl le  H., Gotthardt D.N., Cekauskas A., Manikas M., Mikalauskas S. (2011). The use of high-dose melatonin in liver resection is safe: First clinical experience. J. Pineal Res..

[196] Cardinali D.P., Pandi-Perumal S.R., Niles L.P., Monti J.M., Pandi-Perumal S.R., Sinton C.M. (2008). Melatonin and Its Receptors: Biological Function in Circadian Sleep-Wake Regulation. Neurochemistry of Sleep and Wakefulness.

[197] Dagan Y., Zisapel N., Nof D., Laudon M., Atsmon J. (1997). Rapid reversal of tolerance to benzodiazepine hypnotics by treatment with oral melatonin: A case report. Eur. Neuropsychopharmacol..

[198] Garfinkel D., Zisapel N., Wainstein J., Laudon M. (1999). Facilitation of benzodiazepine discontinuation by melatonin: A new clinical approach. Arch. Intern. Med..

[199] Siegrist C., Benedetti C., Orlando A., Beltran J.M., Tuchscherr L., Noseda C.M., Brusco L.I., Cardinali D.P. (2001). Lack of changes in serum prolactin, FSH, TSH, and estradiol after melatonin treatment in doses that improve sleep and reduce benzodiazepine consumption in sleep-disturbed, middle-aged, and elderly patients. J. Pineal Res..

[200] Kunz D., Bineau S., Maman K., Milea D., Toumi M. (2012). Benzodiazepine discontinuation with prolonged-release melatonin: Hints from a German longitudinal prescription database. Expert Opin. Pharmacother..

[201] Clay E., Falissard B., Moore N., Toumi M. (2013). Contribution of prolonged-release melatonin and anti-benzodiazepine campaigns to the reduction of benzodiazepine and Z-drugs consumption in nine European countries. Eur. J. Clin. Pharmacol..

[202] Leger D., Laudon M., Zisapel N. (2004). Nocturnal 6-sulfatoxymelatonin excretion in insomnia and its relation to the response to melatonin replacement therapy. Am. J. Med..

[203] Zhdanova I.V., Wurtman R.J., Regan M.M., Taylor J.A., Shi J.P., Leclair O.U. (2001). Melatonin treatment for age-related insomnia. J. Clin. Endocrinol. Metab..

[204] Wilson S.J., Nutt D.J., Alford C., Argyropoulos S.V., Baldwin D.S., Bateson A.N., Britton T.C., Crowe C., Dijk D.J., Espie C.A. (2010). British Association for Psychopharmacology consensus statement on evidence-based treatment of insomnia, parasomnias and circadian rhythm disorders. J. Psychopharmacol..

[205] Cardinali D.P., Furio A.M., Brusco L.I. (2011). The use of chronobiotics in the resynchronization of the sleep/wake cycle. Therapeutical application in the early phases of Alzheimer’s disease. Recent Pat. Endocr. Metab. Immune Drug Discov..

[206] Jean-Louis G., von Gizycki H., Zizi F. (1998). Melatonin effects on sleep, mood, and cognition in elderly with mild cognitive impairment. J. Pineal Res..

[207] Peck J.S., LeGoff D.B., Ahmed I., Goebert D. (2004). Cognitive effects of exogenous melatonin administration in elderly persons: A pilot study. Am. J. Geriatr. Psychiatry.

[208] Wade A.G., Ford I., Crawford G., McMahon A.D., Nir T., Laudon M., Zisapel N. (2007). Efficacy of prolonged release melatonin in insomnia patients aged 55–80 years: Quality of sleep and next-day alertness outcomes. Curr. Med. Res. Opin..

[209] Riemersma-van der Lek R.F., Swaab D.F., Twisk J., Hol E.M., Hoogendijk W.J., van Someren E.J. (2008). Effect of bright light and melatonin on cognitive and noncognitive function in elderly residents of group care facilities: A randomized controlled trial. JAMA.

[210] Garzon C., Guerrero J.M., Aramburu O., Guzman T. (2009). Effect of melatonin administration on sleep, behavioral disorders and hypnotic drug discontinuation in the elderly: A randomized, double-blind, placebo-controlled study. Aging Clin. Exp. Res..

[211] Cazzola R., Rondanelli M., Faliva M., Cestaro B. (2012). Effects of DHA-phospholipids, melatonin and tryptophan supplementation on erythrocyte membrane physico-chemical properties in elderly patients suffering from mild cognitive impairment. Exp. Gerontol..

[212] Rondanelli M., Opizzi A., Faliva M., Mozzoni M., Antoniello N., Cazzola R., Savare R., Cerutti R., Grossi E., Cestaro B. (2012). Effects of a diet integration with an oily emulsion of DHA-phospholipids containing melatonin and tryptophan in elderly patients suffering from mild cognitive impairment. Nutr. Neurosci..

